# Optimization of construction safety resource allocation based on evolutionary game and genetic algorithm

**DOI:** 10.1038/s41598-023-44262-9

**Published:** 2023-10-10

**Authors:** Junlong Peng, Qi Zhang, Yue Feng, Xiangjun Liu

**Affiliations:** School of Transportation Engineering, Changsha University of Science and Technology, Changsha, 410114 China

**Keywords:** Civil engineering, Human behaviour

## Abstract

In the construction industry, ensuring the safety performance of a project relies heavily on the effective allocation of safety resources. As the importance of mental health in the construction industry increases, evolutionary game theory can be used to analyze the interaction mechanism of various factors affecting safety performance during the construction phase. The objective of this paper is to construct an analytical model that combines evolutionary game theory with genetic algorithms from the perspective of Leader-Member Exchange Ambivalence. The model aims to quantify and compare the various factors that influence achieving the expected safety state and identify the specific necessary constraints. Initially, we analyzed the relationships among construction site employees, divided them into superiors and subordinates, and established a game model and payoff matrix based on the research background. Next, we introduced genetic algorithms into the model via the replicator dynamic equation for optimization. We adjusted the coefficients of safety risk level, psychological expected return, moral identity, and other factors to simulate various construction site scenarios. Simulation and optimization results indicate that genetic algorithms provide more accurate reference values for safety resource allocation compared to preset or manually assigned values.

## Introduction

The extensive, dynamic, and decentralized characteristics of the construction process determine the prevalence of safety accidents in construction^1,2^. Taking China as an example, in 2020, there were a total of 689 production safety accidents in housing and municipal construction projects nationwide, resulting in 794 fatalities. In terms of safety management in construction projects, accident prevention is an extremely challenging task because of the diverse site environment and the complex relationships among the numerous employees^3^. The various noncompliant behaviors arising from the pursuit of greater profits by the parties involved in construction projects have had a significantly negative impact on safety performance. Despite the numerous innovative practices and intelligent early warning models proposed in recent years to improve safety performance^4–6^, the continuously increasing accident rate proves that there is still room for improvement in safety. Therefore, scholars have adopted various methods and models to measure the safety performance of construction projects^7,8^, many of which focus on the inherent objective environment within the construction site. These indicators are inadequate because, to a large extent, the correlation between human factors and safety accidents is greater than that between the built environment or equipment performance and safety accidents. Therefore, taking a people-centered approach and exploring a more rational path for safety resource allocation, enhancing employees’ safety awareness on construction sites, and improving the efficiency of safety management are all of paramount importance.

Frontline workers, as the direct producers of construction products and the first victims of construction accidents, have long been widely studied by scholars^9–11^. Many specific measures and management models for controlling workers’ unsafe behaviors have been proposed, including safety-related regulations and computer-based methods^12,13^. However, in order to achieve effective management results, previous research has mostly focused on optimizing regulatory mechanisms from the perspective of mandatory measures, and lacks exploration of the interaction mechanisms among employees within the construction site^14^. Although research on construction safety has been ongoing for a long time, in the current era of tremendous change, psychological health in the construction industry seems to be undervalued. As young people in the new era enter the construction industry in large numbers, psychological health issues are particularly prominent in construction safety^15^. Young practitioners in the new era have stronger personal characteristics, are more willing to express their ideas, and have a greater resistance to mandatory punitive measures^16^. Once the attitude of neglect towards construction safety is formed, it is difficult to change and can easily lead to safety hazards.

Once safety hazards exist, the countdown to the occurrence of safety accidents begins^17^. Safety resources constitute the most pivotal investment in construction projects, yet during the actual construction process, safety investments are frequently curtailed or diverted to other areas. The purpose of safety investment is to address latent security risks. Current safety investments generally target overarching strategies and significant mechanisms, which seldom encounter grave issues. Consequently, these investments struggle to assume a pivotal role in real accidents^18,19^. To minimize the impact of safety hazards, the allocation of safety resources should not be limited to the increase of safety personnel or the addition of safety equipment^2^. This approach not only wastes a considerable amount of resources but also proves difficult to yield desirable results^20^. For instance, conflicts between workers and safety managers often arise, and redundant safety equipment can have a counterproductive effect on work progress. In order to better investigate the changes in safety status during the construction process, this paper abstracts the specific participating units as roles of superiors and subordinates that can be transformed into each other^21^. Game theory of evolution is introduced to analyze the behavioral trends of both sides under different conditions. Furthermore, in the game model, the replicator dynamics equation is used as the basis to consider the optimization of safety resource allocation with heuristic algorithms.

The organization of this paper is as follows: in the second section, related literature is summarized; in the third section, the research background is introduced and a model combining evolutionary game theory and genetic algorithms is established; in the fourth section, a practical case is selected for simulation analysis; finally, the conclusion section provides the findings of this study.

## Related work

### Leader-member exchange ambivalence and construction safety climate

Leader-Member Exchange (LMX) is a theory that describes the relationship between a supervisor and their employees^22^. The concept is derived from the Social Exchange Theory, which is a theory that explains how social relationships are formed and maintained based on the exchange of rewards and costs^23^. LMX theory suggests that supervisors form different types of relationships with their employees based on their levels of trust, respect, and mutual obligations^24–26^. These relationships are categorized into two groups: in-group and out-group. In-group members have a closer relationship with their supervisor, which results in more favorable job attitudes and behaviors, such as job satisfaction, organizational commitment, and job involvement. In contrast, out-group members have a more formal relationship with their supervisor, resulting in less favorable job attitudes and behaviors and lower levels of performance. Therefore, LMX is a key variable in understanding how workplace relationships between supervisors and workers can affect employee attitudes, behaviors, and performance^27^. However, the leader’s overly critical care behavior can easily lead employees to perceive conflicts in the superior-subordinate relationship^28^.

LMX ambivalence is a concept that describes a mixed or uncertain attitude that employees may have towards their supervisor^29^. It is characterized by feelings of both positive and negative emotions towards the supervisor, resulting in a conflicted or ambivalent relationship. Research has shown that LMX ambivalence can have negative effects on employee job attitudes and behaviors^30,31^. For example, employees who experience LMX ambivalence may feel less committed to their organization and may have lower levels of job satisfaction. They may also exhibit lower levels of job performance. LMXA can also be applied to construction site workers. Frequent interactions in a harsh hierarchical relationship can cause ambiguity.

When superiors prioritize safety goals and provide adequate information, resources, and support in addressing subordinates’ safety concerns, it can lead to subordinates acting in a safer manner and promoting safety performance in construction projects^32^. In contrast, poor supervisors (who set unclear safety goals) can undermine subordinates’ initiative towards creating a safe environment in construction projects and increase their tendency to violate safety rules^33^. Considering the high-risk nature of the construction industry and the inherent conflicting experiences between safety managers and workers. The existence of high-quality superior-subordinate relationships is necessary to influence workers’ psychological perception towards violating or adhering to safety rules.

The destructive effects of employee conflict psychology have been widely demonstrated. Han^34^ proposed that when there is conflict between superiors and subordinates, employees may experience work stress and a sense of insecurity, which may lead to a lack of work motivation and affect their job performance and productivity. Huang et al.^35^ and Lee et al.^29^ arrived at similar conclusions, stating that conflicts between superiors and subordinates can have a negative impact on employees’ work, resulting in an increase in work anxiety. Employees may feel that they are unable to control their work environment and even feel that they are in an unsafe state. Van Harreveld et al.^36^ confirmed this state, stating that in order to alleviate this sense of insecurity, employees typically adopt defensive coping strategies.

The roots influencing workers’ behavior lie in intrinsic mechanisms such as risk perception, personality traits, and knowledge of safety^37^. Safety Climate is the comprehensive embodiment of an organization’s or workplace’s internal staff members’ perceptions, beliefs, and attitudes towards safety matters and practices. It mirrors the degree of importance employees place on safety issues within the work environment, as well as whether the organization exhibits concern and support for safety^1^. Active engagement of workers in fostering a safety climate serves to alleviate the burden of safety-related stress, while also actively contributing to accident prevention and mitigating losses incurred in accidents^38^. The preceding discussion has elucidated the assertive dispositions of the new generation of construction workers. From this, it becomes evident that the significance of safety climate is accentuated even further. A congruent management approach holds significant positive effects in guiding and fortifying employees’ safety consciousness.

### Optimisation of safety resource allocation

In the past few decades, scholars have made tremendous efforts to reduce the incidence of accidents in construction projects. In addition to conventional methods such as interviews, questionnaires, and data mining, other mathematical models have been employed to study the interactions among stakeholders in the construction industry. Among them, the theory of evolutionary game theory has played an important role in exploring the mechanisms of mutual influence among participating parties. Compared to classical game theory, evolutionary game theory places more emphasis on the dynamics of strategy changes, which is highly compatible with the long-term and temporary nature of construction projects. In the field of construction safety, regulatory mechanisms have long been a hot topic of discussion. The regulatory game involving government participation has been used to test the applicability of China’s construction project supervision system and to demonstrate the effectiveness of the punishment system^39^. Gong et al.^40^ analyzed the decision-making interaction among stakeholders under both static and dynamic supervisory mechanisms to eliminate information asymmetry between the government and contractors in construction projects and improve the safety performance of construction projects. Jiang et al.^41^ further analyzed the game relationship of safety regulatory mechanisms on construction sites from the perspective of the involvement of supervising engineers and pointed out that excessive government regulation could have negative impacts.

However, the parameter settings and assignments in evolutionary game theory are fraught with uncertainty. In other words, at times the parameter assignments in evolutionary game theory may be overly idealized, making it difficult to ensure their universality. Therefore, it is necessary to evaluate a wider range of parameter combinations.

Heuristic algorithms are a type of computational method based on heuristic principles, often used to find approximate optimal solutions or values in large-scale problems^42,43^. For the replication dynamic equation based on evolutionary game theory, there are generally multiple parameters to be solved, making it difficult to accurately calculate the optimal parameter combination. In this case, heuristic algorithms are particularly suitable due to this characteristic. Genetic algorithm is a commonly used heuristic algorithm, which is frequently applied in various aspects of the construction industry, such as labor allocation^44^, resource allocation^45^, and building design^46^.

To address the uncertainty in evolutionary game theory and the difficulty in quantifying psychological factors in safety resource allocation, this study proposes a model that combines evolutionary game theory with genetic algorithm. Based on the interactions between internal stakeholders on construction sites, the model abstracts numerous participating employees into superiors and subordinates, forming game objects. The application of this model in practical cases makes the study more rational and applicable. The main contributions of this paper are as follows: (1) refining the complex interactions between employees on construction sites into a model of bilateral relationships; (2) establishing a new game model in construction sites from the perspective of the conflicts experienced by superiors and subordinates; and (3) optimizing safety resource allocation using evolutionary game theory and genetic algorithm.

## Methodology

### Background description of the studied groups

As the construction process becomes standardized, the involvement of numerous specialized work groups in the management of the construction process becomes imperative. These specialized work groups do not cater to a single entity, and as such, do not receive a uniform directive from a single leader. Additionally, their corporate culture and management methodologies exhibit certain distinctions. The hierarchical structure within the construction site has been significantly blurred, which creates fertile ground for the development of LMX Ambivalence. The existence of cognitive dissonance among stakeholders often leads to a state of confusion and discordance, rendering them vulnerable to negative influences and noncompliance with safety laws and regulations.

The entities participating in a construction project include design firms, survey teams, investment groups, construction companies, supervision companies, and labor subcontractors, among others. Their shared objective is the smooth progress of the project, but during this process, they may encounter special circumstances such as changes in the attitude of the service object. For instance, during the construction preparation stage, the developer is the primary leader. As the project transitions into the build phase, the construction company, with their specialized expertise, becomes the actual controller. The hierarchical relationship can easily become blurred or even reversed. Given this context, we first classify the numerous participating units in a construction project: there are two groups present in a construction project, one referred to as the “Superiors” and the other as the “Subordinates”.

For “Subordinates”, the attitude of their superiors has a significant guiding influence on their actions. If the subordinates perceive themselves as being on the same team as their supervisors and are driven by the pursuit of greater economic benefits, the chances of ignoring laws and regulations will be greatly increased. This could even lead to collusion with other stakeholders and render safety supervision ineffective.

For “Supervisors”, there are many safety hazards in the process of construction projects, which can easily lead to accidents and affect the progress and efficiency of the project. It is necessary to supervise the operation group according to safety standards and legal regulations. However, “Supervisors” often harbor a sense of luck and allow subtle violations by “Subordinates”, even deliberately condoning them for the sake of sharing benefits.

**Figure 1:**
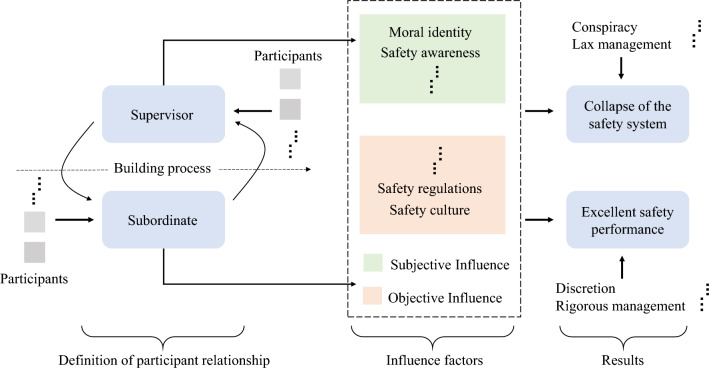
Relationship model.

In this relationship, the “Supervisors” typically refrains from expressing their stance voluntarily to maintain their authority, and instead responds to the actions of the “Subordinates”. “Subordinates” will adjust their behavior based on the response of “Supervisors” within the bounds of bounded rationality if they are not given a clear stance. For instance, if the “Subordinates” engages in minor violations as a test, they will respond to the “Supervisors”’ reaction, either by stopping or colluding, depending on the level of resistance received. The “Subordinates” may intensify its actions, perceiving this as an alignment with its own side, if the “Supervisors” chooses to condone such behaviour. A model of the relationship is shown in Fig. 1. The subjective behavior of a group is difficult to regulate. Thus, it is necessary to establish a neutral mechanism for supervising safety resources, which enables the two stakeholders to choose an ideal security strategy.

**Figure 2:**
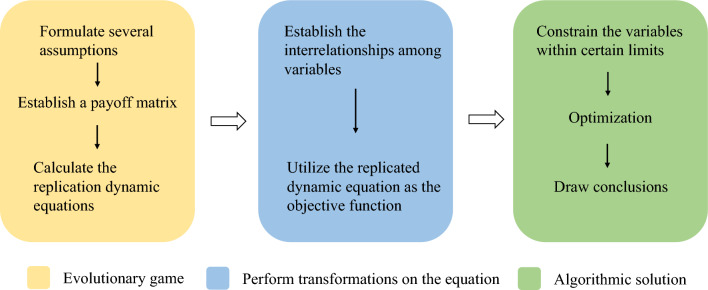
The framework of the research methodology.

In this paper, we achieve the above objective through four steps. (1) Formulate some hypotheses, construct an evolutionary game model, and obtain the replicator dynamics equation. (2) The replication dynamic equation is processed and used as the objective function for subsequent analysis. (3) Constrain the variables of the objective function and use genetic algorithm for optimization. The entire research methodology framework is illustrated in Fig. 2. We employ MATLAB for solving the game model and utilize Python to iteratively implement the genetic algorithm.

### Evolutionary game model

#### Distribution of strategies

In summarizing the analysis of the previous section, it should be noted that psychological conditions have a certain degree of hiddenness and cannot be accurately represented through numerical values. Arbitrarily assigning values will inevitably lead to results that lack authenticity. Therefore, it is necessary to perform correlation processing on the selected indicators. Furthermore, bounded rationality leads to stochasticity in group strategy selection, which may result in risky behavior during expected psychological confrontation.

The classification of players has been described in detail in the previous section. The game is played between two groups in the system: (Supervisors, Subordinates). Consider strategy combinations based on players’ proactive behavior. The set of strategies of the “Subordinates” is (compliance with rules, non-compliance with rules). For “Subordinates”, testing the rules is the first step to gain greater benefits. To demonstrate distinctiveness, it is assumed that “Subordinates”’ testing behavior, once tolerated, will further escalate the scope of damage to gain additional benefits. For managers, while they also prioritize benefits, they bear the primary responsibility for negative consequences. In China’s construction industry, when safety accidents occur, regulators not only face economic penalties but also fear the negative impact on their personal development. Therefore, the “Supervisors”’ strategy set is (strict regulation, collusion).

#### Parameter setting and solution of the payoff matrix

The loss of human life is an unbearable tragedy. However, various reasons(cost-cutting measures, taking shortcuts, and a sense of overconfidence) often exist that lead individuals to make unsafe choices. Table 1 displays the fundamental parameters utilized in the model, including their assumptions and descriptions. These parameters were determined through on-site investigations, social media comments, and expert interviews in engineering safety management. We invited 10 experts in the field of architecture to participate in the study, including two project managers, three senior engineers, two professors who specialize in research related to this field, and three young professionals working in the construction industry. The demographic information of the participants is shown in Table 2. The moderator summarized the report and discussed the applicability of the data with the experts. Afterward, the moderator provided feedback to the experts and repeated this process until a consensus was reached. Setting the range of parameter values is crucial for optimizing safety resource configuration. For ease of computation, we have set the value range of the parameters to (0, 10) and the coefficient value range to (0, 1), with a precision of 0.01. These values represent the relative values of benefits or losses for comparison purposes, rather than representing their actual meanings.

**Table 1:** Settings of the parameters.

Group	Parameter	Revenue	Cost	Meaning
Supervisors	${P_1}$	$\checkmark$		Benefits for supervisors upon successful collusion: Accepting various forms of bribes from subordinates, such as entertainment, money, and goods. Interest stands as the primary motivating factor compelling superiors to embrace risk-bearing^47^.
	${I_1}$	$\checkmark$		Rewards for safety performance.
	${T_1}$		$\checkmark$	“Strict regulation” additional costs: the value of the labor, time, and effort expended in comparison to the “collusion”.
Subordinates	${T_2}$		$\checkmark$	“Compliance with rules” costs: the costs associated with prioritizing safety, referring to the value of the additional labor, time, and effort expended.
	${I_2}$	$\checkmark$		Rewards for safety performance.
	${P_2}$	$\checkmark$		Cost reductions for subordinates in the event of successful collusion: cost savings from “taking shortcuts”, swapping materials, etc. Although realizing cost savings by neglecting adherence to building standards can yield notable benefits for practitioners, it also ushers in significant safety risks^48^.
	${T_3}$		$\checkmark$	“Non-compliance with rules” strategy when being “strict regulation” causes reputational damage: loss of trust in subordinates by supervisors and a decline in leadership within subordinates. Internal leadership within the construction team primarily hinges on the guiding influence of the foremen. Once the workers’ sense of alignment with the foremen wanes, sustaining the foremen’s control over the team becomes challenging^15^.
	*r*		$\checkmark$	Moral identity coefficient: the negative impact of unsafe behaviour on work engagement. The higher the value, the stronger the guilt about the rule-breaking behaviour^49^.
Rule variables	*R*		$\checkmark$	Negative impact on safety responsibility: China’s Regulations on the Management of Construction Work Safety stipulate that all parties involved in the construction have supervisory responsibilities.
	*F*		$\checkmark$	Safety penalties: established in accordance with the safety rules within the construction site and the relevant laws and regulations.
Influencing parameters	$\theta$			The safety level of a construction site: the root cause that affects the overall safety management systemcite^50^. Simplified representation as the likelihood of an accident occurring.
	*W*			Supervisor-subordinate intimacy: The primary basis for judging the likelihood of a subordinate’s perceived success in a collusion. Simplified in this paper as the starting benefit of the “non-compliance with rules” strategy.
	*H*			Security Perception Judgement: The main basis for judging the adoption of collusion strategies by superiors. Simplified in this paper as the starting benefit of a “collusion” strategy.

**Table 2:** Demographic information of the participants.

Expert	Type of unit	Career	Education	Age
Expert1	Construction firm	Project Manager	Bachelor degree	52
Expert2	Construction firm	Project Manager	Bachelor degree	58
Expert3	Engineering Design Institute	Senior engineer	Master degree	36
Expert4	Engineering consulting firm	Consulting Engineer	Master degree	38
Expert5	Construction firm	Senior engineer	junior college	38
Expert6	College	Professor	Doctor degree	45
Expert7	College	Professor	Doctor degree	55
Expert8	Construction firm	Architectural practitioner	junior college	32
Expert9	Construction firm	Architectural practitioner	Bachelor degree	26
Expert10	Construction firm	Architectural practitioner	Bachelor degree	26

The evolutionary game model proposed in literatures^41,49,51^ were utilized to conduct various perspectives of optimization analysis on the implementation pathway of safety performance in the construction industry. There exists a typical 2-player, 2$\times$2 game model, as well as an extended 3-player, 3$\times$3 game model with 8 strategy sets. Taking the 2$\times$2 game as an example, each player can choose one of two strategies: positive or negative. Players receive rewards ($\alpha$) by achieving a common goal, and receive punishment ($\beta$) for betraying each other. If one player chooses a positive strategy and the other chooses a negative strategy, the player who chooses to betray receives a temptation payoff ($\gamma$), while the player who chooses to cooperate receives a neutral payoff ($\delta$). Furthermore, in the literature^52^, it has been proven that in order for the assumptions to be generalized, the following condition must be met: $\gamma> \alpha> \beta > \delta$. In summary, the gains or losses are represented by setting parameters, which include the players’ subjective psychological evaluations and neutral constraint conditions.

However, the subjective psychological states of participants are difficult to quantify directly with numbers. Through the methods presented in literature such as^52,53^, indirect parameters and the relationships between parameters can be used to represent the effects of subjective psychological states. For example, the psychological state of participants can be influenced by the cost-benefit relationship, which can more accurately explain how psychological states guide behavior. Therefore, it is necessary to explain the relationships between the parameters in more detail.

(strict regulation, compliance with rules): When both parties prioritize safety, the safety risk is minimized. As a result, both parties only need to pay regulatory costs and will receive rewards. The supervisors’ expected payoff is ${I_{1}}{ - }{T_1}$, and the subordinates’ expected payoff is ${I_{2}}{ - }{T_{2}}$.

(collusion, non-compliance with rules): When neither party values safety, the associated safety risk is at its highest. In this paper, the subordinate’s moral identity index only affects the initial payoff *W* of the “non-compliance with rules”. The expected payoff for the supervisors is $H + P_1 - \theta R$, while that for the subordinates is $(1 - r)W + P_2 - \theta F$.

When only one party values safety while the other does not, the safety risk exists within the construction site but to a lesser extent than (collusion, non-compliance with rules). As the subject of safety behavior is the subordinate, we set the probability of risk for (strict regulation, non-compliance with rules) to be twice that of (collusion, compliance with rules). In this case, the expected payoffs for the (strict regulation, non-compliance with rules) strategy are as follows: the expected payoff for the supervisors is $-T_{1} -\frac{2}{3} \theta R$, and the expected payoff for the subordinates is $-T_{3} +(1-r)W-\frac{2}{3} \theta F$. In (collusion, compliance with rules) scenario, the expected payoff for the supervisors is $H - \frac{1}{3}\theta R$, and for the subordinates is $-T_{2} - \frac{1}{3}\theta F$. The payoff matrix is presented in Table 3.

**Table 3:** Payoff matrix.

	Strategy	Supervisors	
		Strict regulation *y*	Collusion $1-y$
Subordinates	Compliance with rules *x*	${I_{1}}{ - }{T_1}$, ${I_{2}}{ - }{T_{2}}$	$H-\frac{1}{3} \theta R$, $-T_{2} -\frac{1}{3} \theta F$
	Non-compliance with rules $1-x$	$-T_{1} -\frac{2}{3} \theta R$, $-T_{3} +(1-r)W-\frac{2}{3} \theta F$	$H{ + }{P_{1}} - \theta R$, $(1 - r)W + {P_2} - \theta F$

**Figure 3:**
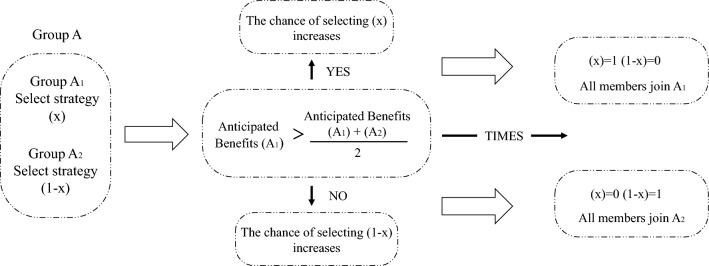
The evolutionary process of strategic selection.

The assumption of bounded rationality is central to the theory of evolutionary game theory. Viewed from an outsider’s perspective, the selection of certain strategies may appear exceedingly absurd. However, these strategies are influenced by players’ psychological expectations of returns and the interactions between various stakeholders with vested interests. Through the iterations of time, previously scorned strategies are more likely to be selected in the future, until they reach the evolutionarily stable strategy(ESS)^54^. As shown in Fig. 3. In other words, when a strategy’s payoff exceeds the average payoff from the previous phase, players will adjust their strategies based on expected returns. Additionally, the speed at which players choose strategies is also an important observed factor, particularly in the context of safety behavior. Safety incidents can result in explosive harm, and the mere thought of them can foreshadow personnel casualties. Safety accidents result in sudden and irreversible harm. Once the thought of unsafe behavior arises, it lays the groundwork for personnel casualties. The increase or decrease in the proportion of strategy selection and its rate of convergence can be analyzed through the replicator dynamic equation.

Based on the parameter relationships of all parties in Table 3 and replicator dynamics, the expected returns of two groups under different sets of strategies can be calculated. The expected payoff for “compliance with rules” is denoted as ${X_x}$, while the expected payoff for “non-compliance with rules” is denoted as ${X_{1{ - }x}}$. The average expected payoff for the subordinates is represented by $\overline{X}$.

1 $$\begin{aligned} {X_x}&= y \cdot ({I_2} - {T_2}) + (1 - y) \cdot \left( - {T_2} - \frac{1}{3}\theta F\right) \nonumber \\&= y \cdot ({I_2} + \frac{{\theta F}}{3}) - {T_2} - \frac{{\theta F}}{3} \end{aligned}$$

2 $$\begin{aligned} {X_{1-x}}&= y \cdot \{ - {T_3} + (1 - r) \cdot W - \frac{2}{3}\theta F\} \nonumber \\&\quad + (1 - y) \cdot \{ (1 - r) \cdot W + {P_2} - \theta F\} \nonumber \\&= y \cdot \left( \frac{{\theta F}}{3} - {T_3} - {P_2}\right) + {P_2} - \theta F - (1 - r) \cdot W \end{aligned}$$

3 $$\begin{aligned} \overline{X}&= x \cdot {X_x} + (1 - x) \cdot {E_{1 - x}}\nonumber \\&= (1 - x) \cdot (1 - r)W - x \cdot {T_2} + (1 - x) \cdot (1 - y){P_2}\nonumber \\&\quad - y \cdot {T_3} + x \cdot y \cdot ({I_2} + {I_3}) + (2x + y - 3)\frac{{\theta F}}{3} \end{aligned}$$

The replicated dynamic equation *f*(*x*) of the subordinates’ behavioral strategy is as follows:

4 $$\begin{aligned} f(x)&= \frac{{dx}}{{dt}} = x \cdot ({X_x} - \overline{X} )\nonumber \\&= x \cdot (1 - x) \cdot \left[ \frac{2}{3}\theta F - \{ {P_2} + {T_2} + (1 - r)W\} \right. \nonumber \\&\quad \left. + y \cdot ({I_2} + {P_2} + {T_3})\right] \end{aligned}$$

Similarly, the expected payoff for “strict regulation” is denoted as ${Y_y}$, while the expected payoff for “collusion” is denoted as ${Y_{1{ - }y}}$. The average expected payoff for the supervisors is represented by $\overline{Y}$.

5 $$\begin{aligned} {Y_y}&= x \cdot ({I_1} - {T_1}) + (1 - x) \cdot \left( H - \frac{{\theta F}}{3}\right) \end{aligned}$$

6 $$\begin{aligned} {Y_{1-y}}&= x \cdot \left( - {T_1} - \frac{2}{3} \theta R\right) + (1 - x) \cdot (H + {P_1} - \theta R) \end{aligned}$$

7 $$\begin{aligned} \overline{Y}&= y \cdot {Y_y} + (1 - y) \cdot {E_{1 - y}}\nonumber \\&= (1 - x) \cdot (1 - y){P_1} + H - x \cdot (H + {T_1}) \nonumber \\&\quad + x \cdot y \cdot {I_1} + (2y + x - 3) \cdot \frac{{\theta R}}{3} \end{aligned}$$

The replicated dynamic equation *f*(*y*) of the supervisors’ behavioral strategy is as follows:

8 $$\begin{aligned} f(y)&= \frac{{dy}}{{dt}} = y \cdot ({Y_y} - \overline{Y} )\nonumber \\&= y \cdot (1 - y)\left\{ \frac{2}{3}\theta R - {P_1} + x \cdot ({I_1} + {P_1})\right\} \end{aligned}$$

#### Model solution

According to Eq. (4), it is evident that $f(x) = 0$ only when $x = 0, 1$, or when *y* equals ${y^*}$. Consequently, the proportion of subordinates who opt for the “compliance with rules” strategy is locally stable. As indicated in Eq. (8), $f(y) = 0$ only when $y = 0, 1$, or when *x* equals ${x^*}$. Therefore, the proportion of Supervisors who select the “strict regulation” strategy is locally stable. Hence, the replicator dynamic system consisting of Eqs. (4) and (8) has five local equilibrium points: *O*(0, 0), *A*(0, 1), *B*(1, 0), *C*(1, 1), and $D({x^*},{y^*})$. Here, ${x^*}$ is expressed by Eq. (9), and ${y^*}$ is expressed by Eq. (10).

9 $$\begin{aligned} x = {x^*}&= \frac{{3{P_1} - 2\theta R}}{{3({I_1} + {P_1})}} \end{aligned}$$

10 $$\begin{aligned} y = {y^*}&= \frac{{3({P_2} + {T_2} + W - rW) - 2\theta F}}{{3({I_2} + {P_2} + {T_3})}} \end{aligned}$$

In accordance with the method proposed by Friedman, the Evolutionarily Stable Strategy (ESS) for a system of differential equations can be obtained by analyzing the local stability of the Jacobian matrix of the replicator dynamic system^55^. The equations are formulated by Eqs. (4) and (8), and the Jacobian matrix is represented by *J*.

11 $$\begin{aligned} J = \left[ \begin{array}{cc} {\frac{{\partial f(x)}}{{\partial x}}}&{} {\frac{{\partial f(x)}}{{\partial y}}}\\ {\frac{{\partial f(y)}}{{\partial x}}}&{} {\frac{{\partial f(y)}}{{\partial y}}} \end{array}\right] \end{aligned}$$

where

12 $$\begin{aligned}&\frac{{\partial f(x)}}{{\partial x}} = \frac{{(1 - 2x)}}{3} \cdot \left\{ \begin{array}{l} 2\theta F - 3({P_2} + {T_2} + W - rW)\\ + 3y\cdot ({I_2} + {P_2} + {T_3}) \end{array}\right. \end{aligned}$$

13 $$\begin{aligned}&\frac{{\partial f(x)}}{y} = x \cdot (1 - x) \cdot ({I_2} + {P_2} + {T_3}) \end{aligned}$$

14 $$\begin{aligned}&\frac{{\partial f(y)}}{x} = y\cdot (1 - y)\cdot ({I_1} + {P_1}) \end{aligned}$$

15 $$\begin{aligned}&\frac{{\partial f(y)}}{y} = \frac{{(1 - 2y)}}{3} \cdot \left\{ \begin{array}{l} - 3{P_1} + 2\theta R\\ 3x\cdot ({I_1} + {P_1}) \end{array}\right. \end{aligned}$$

If the equilibrium point satisfies the two conditions of $DetJ > 0$ and $TrJ < 0$, then the equilibrium point is a locally asymptotically stable fixed point of the evolutionary dynamic process, corresponding to evolutionary stability. The stability analysis of the equilibrium point using the local stability analysis method of the Jacobian matrix yields the results shown in Table 4. The conventional Jacobian matrix local stability analysis method fails to determine the stability of $D({x^*},{y^*})$ since $TrJ=0$. Therefore, the differential analysis method is employed for assessment. By taking the partial derivatives of Eqs. (4) and (8) with respect to *y* and *x* respectively and substituting the coordinates of point $D({x^*},{y^*})$, it is observed that both *df*(*x*)/*dy* and *df*(*y*)/*dx* values are greater than 0. Thus, it can be concluded that $D({x^*},{y^*})$ is an unstable point.

**Table 4:** Equilibrium point stability analysis.

Equilibrium point	Constraint conditions	
	$DetJ > 0$	$Tr < 0$
*O*(0, 0)	$\frac{1}{9}(2\theta R - 3{P_{1}})(2\theta F - 3({P_{2}} - rW + {T_{2}} + W)) > 0$	$\frac{2}{3}\theta (F + R) - {P_{1}} - {P_{2}} + (r - 1)W - {T_{2}} < 0$
*A*(0, 1)	$\frac{1}{9}(3{P_{1}} - 2\theta R)(2\theta F + 3{I_{2}} + 3((r - 1)W - {T_{2}} + {T_{\textrm{3}}})) > 0$	${I_2} + {P_1} + \frac{2}{3}\theta \left( {F - R} \right) - {T_2} + {T_3} + \left( { - 1 + r} \right) W < 0$
*B*(1, 0)	$- \frac{1}{9}(3{I_{1}} + 2\theta R)(2\theta F - 3({P_{2}} - rW + {T_{2}} + W)) > 0$	$\frac{2}{3}\theta (R - F) + {I_{1}} + {P_{2}} - rW + {T_{2}} + W < 0$
*C*(1, 1)	$\frac{1}{9}\left( {3{I_1} + 2\theta R} \right) \left( {3{I_2} + 2\theta F + 3\left( { - {T_2} + {T_3} + \left( { - 1 + r} \right) W} \right) } \right) > 0$	$- {I_1} - {I_2} - \frac{2}{3}\theta \left( {F + R} \right) + {T_2} - {T_3} + W - rW < 0$

The numerical results of replicated dynamic equations display the direction and rate of the players’ strategy evolution. Hence, heuristic algorithms can be utilized to optimize. Our objective is to maximize *f*(*x*) and *f*(*y*) at a certain scale to encourage both players to adopt strategies that prioritize safety within a given range of parameter settings.

### Genetic algorithm

Genetic algorithm is a heuristic optimization technique inspired by the biological process of natural selection and genetic inheritance. It involves the representation of the problem solution as a population of individuals, where each individual is evaluated based on a fitness function. The fittest individuals are selected for reproduction, and through crossover and mutation operations, their traits are passed on to the next generation of individuals, creating a new population. This process continues until an optimal solution is reached or a termination criterion is met. Genetic algorithms are widely used in many fields, including engineering, economics, and biology, where complex problems require efficient solutions.

**Figure 4:**
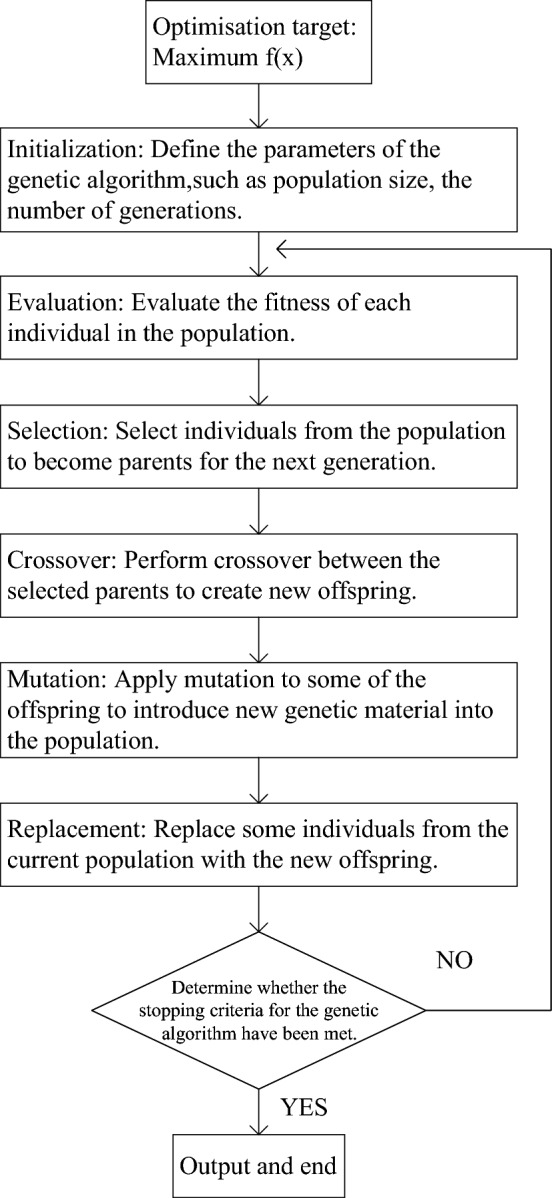
Flowchart of genetic algorithm.

In this paper, the selection of strategies for supervisors is primarily dependent on the actions of their subordinates. It is appropriate to utilize a conventional genetic algorithm to optimize the replicated dynamic equation *f*(*x*) of the subordinates independently. Figure 4 illustrates the optimization process of the traditional genetic algorithm. The critical steps are explained as follows.

(a) Coding and initialization

**Figure 5:**
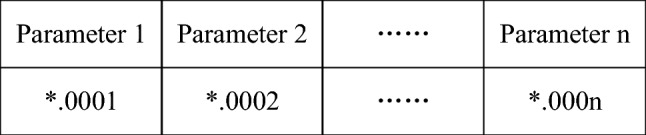
Individual gene encoding.

There are various encoding methods for genetic algorithms, such as binary encoding, Gray encoding, floating-point encoding, cascading encoding for multiple parameters, and multiparameter crossover encoding. For the sake of comparison, there was no requirement for the breadth of the range when discussing the parameter range of variables in the previous section. The unavoidable need for high-precision decimals arises. Therefore, in this section, the floating-point encoding method is utilized. The higher the number of bits in the floating-point representation, the greater the accuracy of the genetic algorithm. Correspondingly, the computational complexity will also be enormous. After taking all factors into account, it was found that four-digit floating-point numbers are sufficient to solve the problems in this section and yield relatively accurate results. As there are eight variables to be solved in this section, each individual’s genotype is represented by eight floating-point numbers. The gene encoding for a single individual is illustrated in Fig. 5. In order to prevent the fitness function from being less than 0, the fitness function is set as $f(x) - f(x)_{min}$. The population size *N* is set to 1000, the maximum number of iterations *Maxgen* is 300, the mutation probability *pm* is 0.2, and the crossover probability *pc* is 0.9.

(b) Selection and Crossover

In this stage, superior individuals are selected while inferior ones are eliminated. After a long period of development, many selection methods have been devised for genetic algorithms. In this section, the well-established roulette wheel selection method is utilized. Individuals are randomly selected using the roulette wheel method based on their fitness values. The higher the fitness value of an individual, the greater their likelihood of being selected. The specific steps are shown as follows:

1. Calculate the total fitness of the population $F = \sum \limits _{i = 1}^n {{f_i}}$.
2. Calculate the ratio of each individual’s fitness to the total fitness, thereby obtaining their probability of selection ${p_i} = \frac{{{f_i}}}{F}$.
3. Combine the probabilities of selection for all individuals into a roulette wheel.
4. Spin the roulette wheel and randomly select an individual. Generate a random number $r \in \left[ {0,1} \right]$. If ${p_i}< r < {p_{i + 1}}$, the selected individual *i* will enter the next generation population. Repeat this process until the population reaches a certain size.

**Figure 6:**
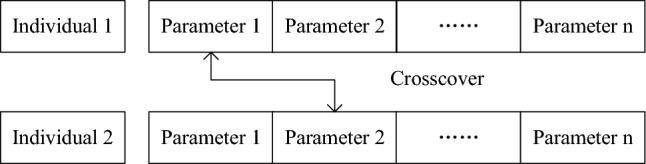
Case of crossover.

The crossover operation mimics the genetic recombination phenomenon in nature. During the crossover process, if two identical individuals’ chromosomes are selected for crossover, then another chromosome is randomly selected from a different, random individual. Once the values of the two selected chromosomes are exchanged, the crossover process is complete. An illustration of the crossover process is shown in Fig. 6.

(c) Mutation

By simulating genetic mutations, diversity is introduced into the population to prevent premature convergence to suboptimal solutions. After chromosome crossover, each chromosome has a 0.2 chance of mutating. Mutations modify one or more genes of the chromosome, with the magnitude of the changes decreasing as the number of iterations increases. By gradually reducing the magnitude of the changes introduced by mutations, the algorithm is more likely to converge towards the optimal solution while maintaining diversity in the population.

(d) Output

Compare the currently best solution with the previous best solution obtained from the entire population. Retain the individual with the highest fitness and repeat the process. When the maximum number of iterations is met, terminate the algorithm and obtain the best individual.

## Case analysis

The scope of construction projects is extremely broad, and the model established in this paper cannot be applied to all construction projects. In order to make the proposed method in this paper more informative and inspiring. We consider a complex project, refer to the safety risk calculation in literature^50^, and apply our proposed method for optimization. During this process, some additional constraints may arise.

**Figure 7:**
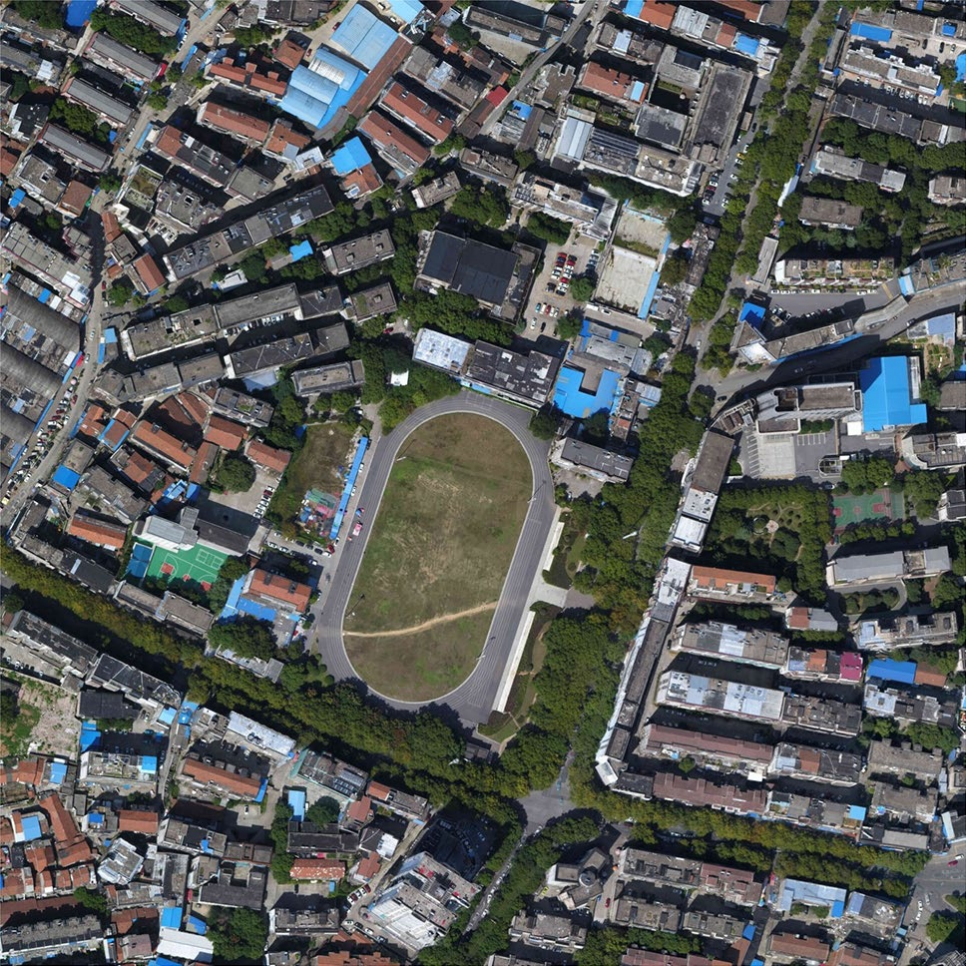
The aerial view of case 1.

The construction project for the expansion and renovation of the Yueyang Workers’ Cultural Palace and Dongfeng Square includes the following elements: a nine-story Workers’ Cultural Palace, a standard sports field, a two-level underground parking lot, and supporting facilities such as roads, plazas, surface parking lots, landscaping, and outdoor pipelines. The project is located in the Dongting New Town area of Yueyang City, with a planned area spanning from Yunmeng Road in the east to Dongting Lake in the west, from Balin West Road in the north to Nanjingang in the south, covering a total land area of 363.8 hectares. The total land area of this project is 48,765.05 square meters, with a net land area of 37,845.33 square meters. Dongfeng Square and the Yueyang Workers’ Cultural Palace are located in the core area of the old city in Yueyang, and are currently the only public cultural and sports activity center in the western part of the urban area, attracting significant attention from the community. The aerial view of the project is illustrated in Fig. 7. The safety risk level of this project $\Theta = 1.805686827$.

In order to enhance the applicability of the model proposed in this article to the corresponding real-world construction projects, we also conducted an investigation into the workforce engaged in this project. The resident workforce for this project comprises 268 individuals, with 250 males and 18 females. Notably, individuals above the age of 50 constitute 55% of this demographic. Operating as a government redevelopment endeavor, the project boasts a relaxed timeline, ample financial resources, and proximity to the urban center. This advantageous context results in diminished work-related pressures and elevated safety standards. A comprehensive safety regimen is upheld on this project, including monthly safety training sessions and frequent safety drills. As a result, the safety climate within the project is notably robust.

Based on the project characteristics and expert evaluation, the basic construction project data and initial parameters are shown in Table 5. Given the multitude of risks and uncertainties inherent in the construction phase of a construction project, place great emphasis on safety management during the construction phase, communication between the two sides deepens as the project progresses. Therefore, the probabilities of adopting positive strategies are $x=0.2$ and $y=0.5$, respectively. In order to visually identify the effects of different parameter changes on the evolutionary results, the number of repeated simulations is considered the number of game iterations, denoted as *t*. The project risk coefficient is a key indicator that determines safe behavior and also has a certain impact on the assignment of various indicators. In addition, the determination of psychological indicators is rather ambiguous, and we adopt a wide-range search to observe the impact of psychological indicators on strategy selection.

**Table 5:** Original design parameters.

Parameter	Initial value	Range
*F*	7.5	(5, 10)
${P_2}$	3	(0, 5)
${T_2}$	2	(1, 3)
*r*	0.5	(0.3, 0.6)
*W*	4	(0, 5)
${I_2}$	5.5	(5, 7)
${T_3}$	6	(5, 7)
*R*	7.5	(5, 10)
${P_1}$	5	(4, 8)
${I_1}$	5.5	(5, 7)
$\theta$	0.6	(0, 1)

### Analysis of the impact of risks, penalties on both players

**Figure 8:**
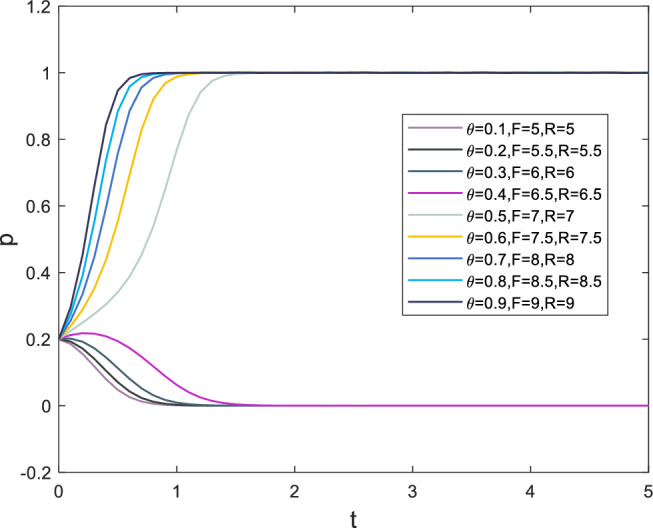
Evolution results of x.

**Figure 9:**
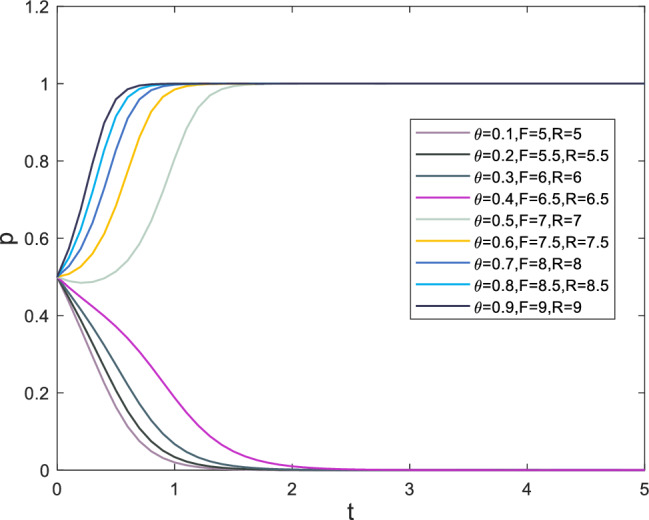
Evolution results of y.

The safety risk factor, denoted as $\theta$ in the case, forms the basis of the initial parameter settings. In this paper, the safety risk factor primarily affects the punishment of both players following a security incident. Correspondingly, the penalties vary with changes in the safety risk factor. The punishments for superiors and subordinates resulting from safety incidents are denoted as *F* and *R*, respectively. The evolutionary process of strategy selection for supervisors and subordinates is displayed in Figs. 8 and 9, respectively, as the safety risk factor $\theta$ changes.

Based on the information presented in Fig. 8, it can be observed that when the safety risk factor exceeds 0.5, the punishment for safety incidents has already reached a high level, causing the subordinates’ strategy selection rate for *x* to gradually increase towards 1. Despite the initial probability of selecting $x=0.2$, indicating that the probability of subordinates choosing to break the rules is much higher than following them, the evaluation of gains tends to be conservative in high-pressure situations. The situation for supervisors is similar, with the rate at which *y* tends towards 1 increasing as the safety risk factor exceeds 0.5.

### Analysis of the impact of psychological factors on subordinates

**Figure 10:**
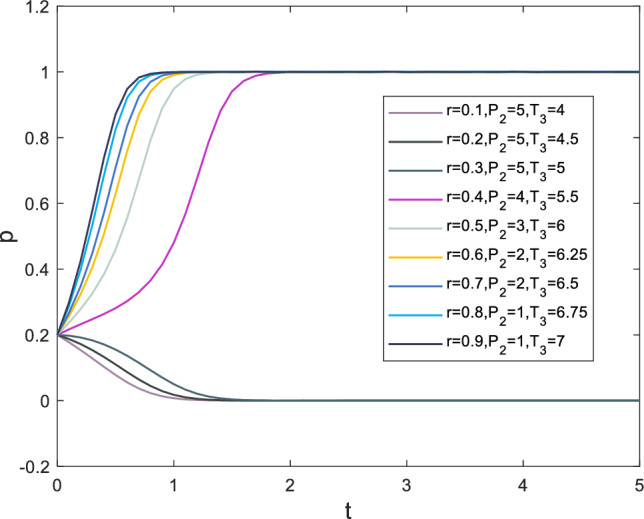
Evolution results of x.

The strategy choices made by subordinates are a crucial consideration for supervisors strategy choices, and can even be referred to as a response. Additionally, as the main executor of the construction process, subordinates possess a greater perception of safety incidents than supervisors. This section provides a separate analysis of subordinates. Subjective emotional factors that influence subordinate behavior primarily include the moral identity coefficient *r*, the initial expected benefits of negative strategies $P_2$, and reputation loss $T_3$. Moral identity represents the employees’ aversion to negative actions toward themselves or others. When an employee has a higher moral identity, the negative effect of their negative behavior on their own emotions is stronger^56^. In this paper, the moral identity coefficient primarily affects the initial psychological benefits of choosing negative strategies. Moreover, as a crucial criterion for measuring employee behavior standards, moral identity also influences an employee’s regard for expected benefits $P_2$ and their own reputation $T_3$. As the moral identity coefficient increases, the numerical value of expected benefits $P_2$ decreases, while the numerical value of reputation loss $T_3$ increases. The evolutionary process of subordinate strategy choices is shown in Fig. 10. Based on the information displayed in Fig. 10, it can be observed that when the safety risk coefficient exceeds 0.4, the speed at which subordinate strategy choice *x* tends toward 1 gradually increases. The presence of moral identity also manifests in employees’ resistance to temptation, which is highly effective in improving safety performance. Therefore, enhancing employees’ moral identity is of paramount importance in safety management.

### Analysis based on genetic algorithm

As stated above, under certain constraints, both players in the game will tend towards the strategy combination we expect. The strategy combination represented by the (strict regulation, compliance with rules) equilibrium point at *C*(1, 1) can ensure the highest level of safety factor. However, it is not an easy task to keep both parties highly vigilant about safety all the time. Safety managers need to pay a great cost to maintain the safety status, and the relaxed attitude towards the safety status persists because the benefits of maintaining the security status cannot match the cost.

**Figure 11:**
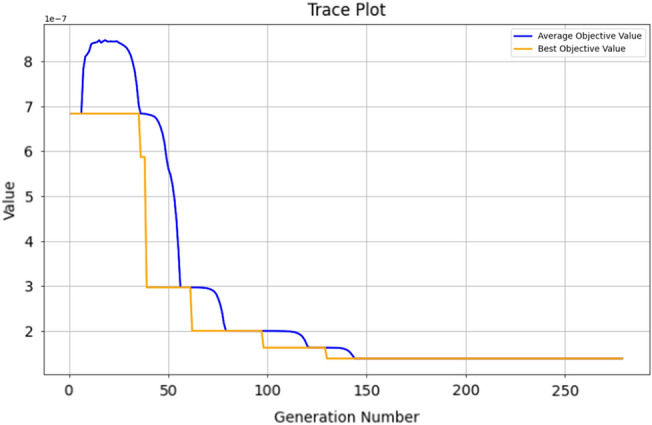
Process curve of the genetic algorithm optimization for minimum value.

**Figure 12:**
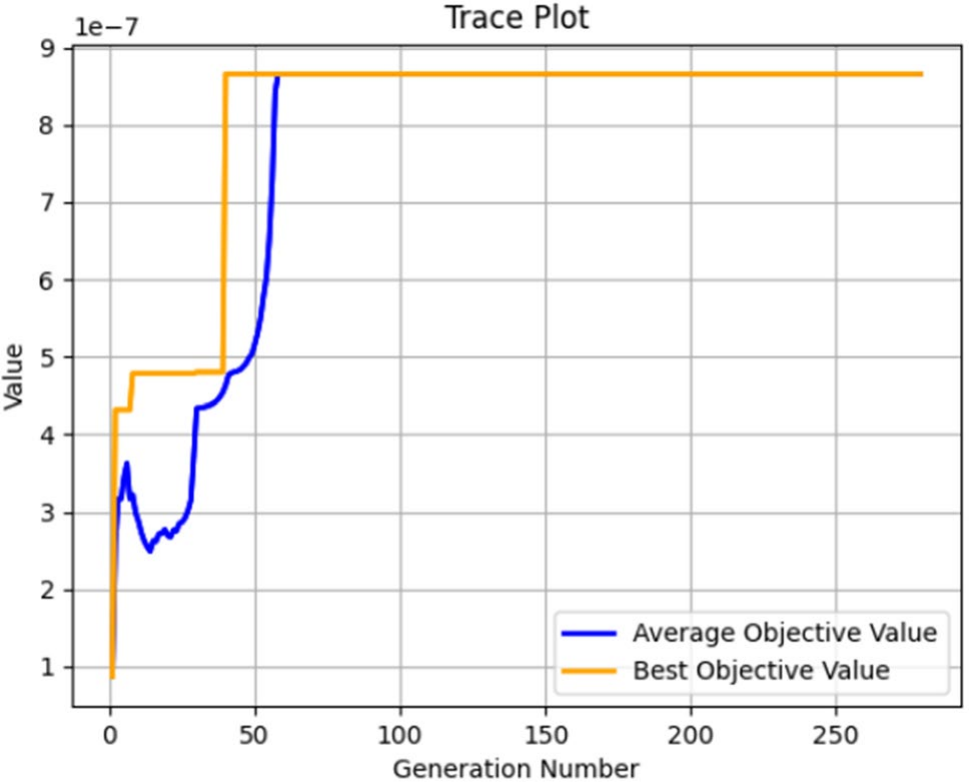
Process curve of the genetic algorithm optimization for maximum value.

In light of this situation, we have utilized genetic algorithms to optimize safety configurations, aiming to achieve a relatively reliable level of safety at the lowest possible cost. At a relatively small scale, we compared the optimization of the minimum and maximum values of the dynamic equation *f*(*x*), ultimately resulting in a secure configuration with a tendency towards 1. The optimization comparison data is presented in Table 6, while Fig. 11 illustrates the process of optimizing the minimum value of *f*(*x*), and Fig. 12 depicts the process of optimizing the maximum value of *f*(*x*).

**Table 6:** Comparative analysis with genetic algorithm.

Parameter	Initial	$f(x)_{max}$	$f(x)_{min}$
*F*	7.5	8.0834	5.3883
${P_2}$	3	4.9529	4.8657
${T_2}$	2	2.8272	2.4375
*r*	0.5	0.3164	0.3066
*W*	4	4.6138	3.8776
${I_2}$	5.5	5.0701	5.3664
${T_3}$	6	5.3581	5.4276

**Figure 13:**
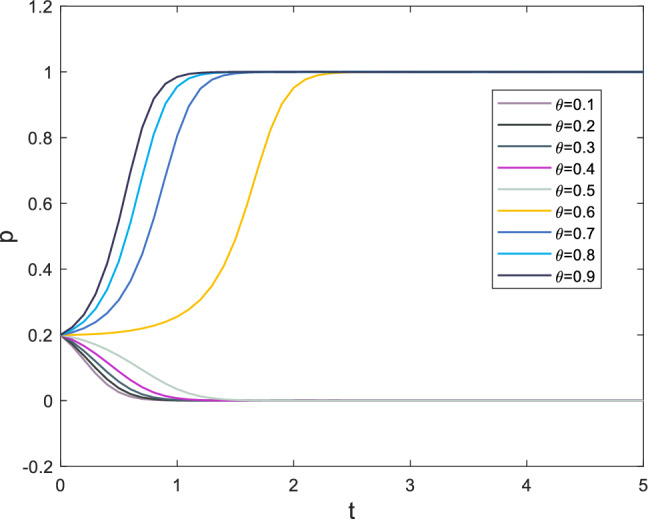
Evolution results of x(min).

**Figure 14:**
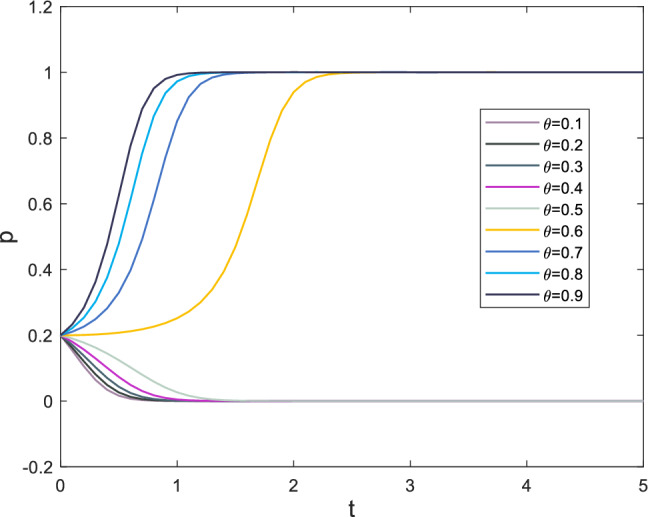
Evolution results of x(max).

On this basis, we modify the safety risk coefficient $\theta$ to observe the evolution of subordinate strategy choices. The evolutionary process is depicted in Figs. 13 and 14. Their evolutionary trends show no significant differences, which is consistent with our expectations. The main differences in safety resource allocation are found in safety punishment *F*, which is largely due to the inhibitory effect of the safety risk coefficient on safety punishment.

## Conclusion

We aim to integrate evolutionary game theory with genetic algorithms to optimize and verify secure resource allocation. Our goal is to identify the optimal allocation of safety resources and specific constraints that promote cooperative behavior, group cohesion, and safety awareness within a construction site, rather than analyzing the expected strategy combinations in the game system. During the construction phase, the dynamic adjustment of safety behavior between superiors and subordinates is a crucial factor that affects safety performance. In construction projects, there are numerous procedures that involve hazardous factors and last for a certain period of time. Therefore, the adjustment of safety behavior is a long-term and ever-changing process, rather than a static choice. The main purpose of evolutionary game theory is to examine the strategic changes of a group. On one hand, individuals learn the optimal strategy by observing and imitating the strategies of other individuals. On the other hand, the group tends to converge to a stable strategy at an evolutionary stable point, which facilitates the observation of overall trends in change.

A drawback of evolutionary game theory is the uncertainty involved in assigning parameters, which makes the design of resource allocation highly inconvenient. In this paper, we propose a method that combines genetic algorithms with evolutionary game theory. By utilizing the characteristics of replicator dynamic equations and specifying the range of numerical values, we optimize the assignment of parameters using heuristic algorithms.

To accurately describe the influencing factors of safety behavior within a construction site, we propose a two-sided evolutionary game model that incorporates both superiors and subordinates. We introduce LMX Ambivalence theory to condense the game system between the two sides into the subordinate’s evolution, enabling better optimization using genetic algorithms. Furthermore, we tested several key parameters, including safety risk level, psychological expected benefit, and expected reputational loss. Simulation results show that there are differences in the ESS under different circumstances, and safety risk level was found to have a significant impact on overall psychological expectation. Moreover, safety penalties did not show a significant effect under the suppression of safety risk level, which was not given sufficient attention in previous studies.

External factors such as safety risk level and moral identification, as well as personal psychological traits, can influence the key parameters and initial ratios of strategies in the game. In safety management, we should fully consider the psychological guidance of employees and the construction of a safety culture within the construction site, rather than only making up for it after a safety accident has occurred. And, investment in this aspect of safety should be strengthened.

There are still some limitations to the method proposed in this paper. We do not intend to analyze which strategy combinations can achieve excellent safety performance, as this is an obvious outcome. Instead, we extract the optimal safety resource allocation from the results of the evolutionary game theory applied to safety management proposed widely above.

However, factors that affect the allocation of safety resources have not been fully incorporated. Due to the gradual modernization of construction sites and the constant emergence of new changes, the differences among various building sites are significant, making it difficult to achieve effective verification through a single model. Future research may explore how other factors influence the evolutionary outcomes, particularly the impact of social opinion on the safety behavior of participating organizations. For example, the construction miracles of Huoshenshan and Leishenshan. One potential future direction is to examine the conditions under which the roles of superiors and subordinates can be interchanged, and whether unexpected events should be taken into account. In addition, it is also necessary to collect more data on building sites through surveys or on-site investigations.

## Data Availability

The datasets used and/or analysed during the current study available from the corresponding author on reasonable request.
